# Is mindfulness protective against PTSD? A neurocognitive study of 25 Tsunami disaster survivors

**DOI:** 10.1186/s12952-016-0056-x

**Published:** 2016-07-20

**Authors:** Christina Hagen, Lars Lien, Edvard Hauff, Trond Heir

**Affiliations:** Department of mental health and addiction, University of Oslo, Oslo, Norway; Norwegian National Advisory Unit on Concurrent Substance Abuse and Mental Health Disorders, Hospital Innlandet Trust, Hedmark, Norway; Department of public health, Hedmark University of Allied Science, Hedmark, Norway

**Keywords:** Trauma, Stress, Trait mindfulness, Tsunami, Survival

## Abstract

**Background:**

It has been suggested that mindfulness is a protective factor that buffers individuals from experiencing severe posttraumatic stress following exposure to a trauma.

We aimed to examine the association between dispositional (trait) mindfulness and posttraumatic stress in individuals who had been exposed to the trauma of a natural disaster.

**Method:**

A disaster group (*n* = 25) consisting of Norwegian tourists who survived the 2004 South East Asian tsunami at a location with high mortality rates was recruited. Dispositional mindfulness and posttraumatic stress were measured with the Five-Facet Mindfulness Questionnaire and the Impact of Event Scale-Revised Version, respectively.

**Results:**

There was no significant association between mindfulness and posttraumatic stress. Moreover, there were no significant associations between posttraumatic stress and the mindfulness sub-facets of observing, acting with awareness, non-judging, and non-reacting. However, there was a significant positive correlation between the descriptive factor of mindfulness and IES-R total. There were no significant linear correlations between the five sub-facets of mindfulness and the three categories of posttraumatic symptoms, intrusion, avoidance and hyper-arousal.

**Conclusions:**

Our findings do not indicate a relationship between dispositional mindfulness and posttraumatic stress levels after exposure to a trauma, except for the descriptive sub-facet of mindfulness and here the correlation is positive and not negative as would be expected if mindfulness is a protective factor for posttraumatic stress. Future studies should investigate the relationship between mindfulness and posttraumatic stress while accounting for factors such as trauma history, type of trauma, and individual differences in traumatic stress reactions.

## Background

Mindfulness (MF) has been defined as bringing one’s complete attention to the present experience on a moment to moment basis [1]. In Western psychology, MF is defined as the intentional and non-judgmental allocation of attention to the present moment [2]. The first clinical handbook of MF [3] noted that despite slight variability in definitions, the core components of MF involve coming into contact with the present moment and observing that moment in a non-judgmental manner. MF training has been proposed as a relevant treatment for traumatised individuals because it encourages acceptance rather than avoidance symptoms; furthermore, the need for research on the utility of MF for trauma survivors has been discussed previously [4].

Posttraumatic stress reactions of avoidance and hyper-vigilance are characterised by incomplete perceptions of the environment that include the misreading of potentially threatening situations and difficulties in accurately labelling one’s own feelings [5]. One of the goals of MF practice is to facilitate an individual’s ability to become aware of experiences in the present moment to enable that individual to functionally engage in life [6]. MF increases psychological awareness and flexibility when responding to emotional experiences [4].

Dispositional or trait MF is defined as the capacity to deliberately attend to present experience without judgment [7]. It has been suggested that the construct of MF consists of various aspects, such as observing, describing, acting with awareness, non-judging, and non-reacting [8]. The observing aspect of MF indicates the tendency by which an individual observes his/her inner life and surroundings, while the describing aspect indicates an individual’s ability to describe his/her feelings. The acting with awareness aspect indicates the tendency of an individual to act with awareness rather than distraction. The non-judging aspect refers to an individual’s tendency to refrain from judging one’s experience and instead relate to that experience with acceptance, and the non-reacting aspect indicates an individual’s tendency to avoid reacting excessively to his/her inner experience [7].

A study by Vujanovic, Youngwirth, Johnson, and Zvolensky [9] analysed the incremental predictive validity of MF-based processes in relation to the severity of the posttraumatic stress symptoms among individuals without axis I psychopathology. The acting with awareness and the accepting without judgment sub-scales were significantly negatively correlated with posttraumatic stress symptom outcomes; the magnitudes of these correlations were small to moderate. Neither the observational nor the descriptive sub-factors of MF were significantly associated with global posttraumatic stress symptoms or the re-experiencing or avoidance symptom clusters. The descriptive MF sub-scale was significantly associated with hyper-arousal symptoms, but the observational sub-scale was not associated with these symptoms.

MF has been associated with reduced symptoms of posttraumatic stress among urban fire-fighters [10]. In a review article, Thompson, Arnkoff, and Glass [11] suggested that trait MF is associated with greater psychological adjustment following exposure to trauma and that experiential avoidance, persistent dissociation, and coping strategies involving emotional disengagement are associated with greater posttraumatic stress severity and related psychopathology.

Dispositional MF has been shown to predict symptoms of posttraumatic stress, and has been seen as a protective factor that buffers individuals against the experience of severe posttraumatic stress [12]. Studies that focus on the potential relationship between MF and posttraumatic stress in clinical and non-clinical samples with different trauma backgrounds are needed.

## Methods

### Aim

The aim of this study was to investigate the relationship between MF and posttraumatic stress in a group with first-hand experience with a natural disaster. We hypothesised that MF would be a protective factor against the symptoms of posttraumatic stress and that there would be a negative relationship between MF and posttraumatic stress.

### Participants

Participants were recruited from an interview study of Norwegian disaster survivors who experienced the 2004 South Asian tsunami in Khao Lak, Thailand [13]. Norwegian tourists staying in Khao Lak were severely affected by the tsunami [14]. Of the 84 Norwegians who perished in the disaster, 68 were staying in Khao Lak. According to registry information from the Norwegian police, there were 82 Norwegian adults who survived in Khao Lak. 63 of these adults participated in the interview study two years after the disaster. For the present study, the same participants were contacted over the phone and asked whether they would be willing to participate in this study. Of these 63 individuals, 3 could not be reached, 2 had died, and 25 agreed to participate. Of these 25 disaster survivors, 13 had been caught by the waves, 6 had been touched or chased by the waves, and 6 reported no direct exposure to the waves. Most of the participants, including all participants who had not been directly exposed to the waves, reported strong witness experiences, such as observing seriously injured individuals, dead bodies, or abandoned children. Five participants reported that a close family member had perished in the tsunami; three participants had lost their husbands; and three participants had lost one or more of their children. The gender distribution in the studied group was 9 males and 16 females. The mean age of the participants in the study was 47.0 years. Intelligence measured by Wechsler Adult Short Intelligence Test (WASI) was one standard deviation above average. Participant characteristics are shown in Table 1.

**Table 1:** Participant characteristics expressed as the means (standard deviations)

	Disaster group (*n* = 25)
Age	47.96 (10.76)
Years of education	15.30 (1.98)
WASI	114.72 (8.82)
IES-R	12.88 (12.90)

Exclusion criteria included serious medical or neurological illness and non-functional Norwegian language skills. Serious medical or neurological illness was defined as a diagnosed medical condition in the somatic or neurological area affecting the brain and/or cognitive functioning.

The fact that 33 participants from the first interview study did not want to participate in this study might affect our results. We want to emphasize that our results were based on data from less than half of the participants in the original interview study. It is possible that individuals suffering from more of posttraumatic stress did not want to participate because of reasons of trauma-related avoidance, intrusion or arousal. Other reasons affecting the decision not to participate, may be physical health, geographical distance, work and family factors, fatigue and cognitive factors, as well as desire or lack of desire to contribute to the research field. Participants were reimbursed for travel expenses and lost income for participation time, in order to minimize the effect of these factors on the decision whether to participate or not. The first interview study was conducted more recently after the tsunami than our study, but this is not likely to have any major implications for our study, since the studies are not related to each other in the way that variables from the first study were used in our study. Our results might have been different though, if our study would have been done closer in time to the tsunami.

### Measurements

#### The Five-Facet Mindfulness Questionnaire (FFMQ)

The Five-Facet Mindfulness Questionnaire [7] was used to measure dispositional (trait) mindfulness. The questionnaire was developed via a factor analysis of a combined pool of items from five other mindfulness questionnaires. The FMMQ consists of 39 items that examine overall trait mindfulness and the mindfulness factors of observing, describing, acting with awareness, non-judging, and non-reacting. Respondents rate each item on a five-point Likert scale that ranges from “*never or very rarely true*” to “*always or almost always true*”. Baer et al. [7] reported adequate to good internal consistencies (ranging from 0.72 to 0.92) for each of the scales. The FFMQ has been validated in several countries [7, 15–19], including Norway [20].

#### The Impact of Event Scale-Revised Version (IES-R)

The Impact of Event Scale-Revised Version [21] was used to measure the symptoms of posttraumatic stress. The scale consists of 22 items with the following three subcategories of PTSD symptoms: re-experiencing (intrusion), avoidance, and arousal. The IES-R has been translated into many languages; it has internal consistency coefficients ranging from 0.80 to 0.91 and test-retest reliabilities ranging from 0.52 to 0.86 [22]. There are other instruments targeting symptoms of posttraumatic stress, such as the Norwegian version of the Posttraumatic Stress Disorder Checklist (PCL) [23]. We chose the IES-R because it is based on the IES (Impact of Event Scale) that is the first and one of the most widely used self-report measures to assess psychological responses to a traumatic stressor [24]. We were also interested in the subscales of intrusion, avoidance and hyper-arousal captured by the IES-R.

#### The Wechsler Adult Short Intelligence Test (WASI)

Intelligence was measured with the Wechsler Adult Short Intelligence Test (WASI) [25]. The WASI is a standardised test that yields the three traditional verbal, performance, and full scale IQ scores. The verbal IQ score is assessed with two measures; specifically, the Vocabulary subtest measures word knowledge, verbal concept formation, and the fund of knowledge, and the Similarities subtest measures verbal reasoning and concept formation. The performance IQ score is based on the following two different types of performance measures: the Matrix Reasoning test measures visual information processing and abstract reasoning skills, and the Block Design test measures the ability to analyse and synthesise abstract visual stimuli, nonverbal concept formation, visual perception and organisation, simultaneous processing, visual-motor coordination, learning, and the ability to separate figures and grounds in visual stimuli.

### Procedures

Written informed consent was obtained from all participants. Information on the study and the option of withdrawing was provided verbally and in writing. In accordance with the ethical standards set forth in the 1964 Declaration of Helsinki, this study was approved by the Regional Ethics Committee and the relevant committees for patient integrity. The participants spent between five and six hours on one day filling out questionnaires and undergoing individual neurocognitive testing. The results of the latter tests will be reported elsewhere. All testing was performed by the same test administrator in a one-on-one setting.

### Statistical analysis

**Fig. 1:**
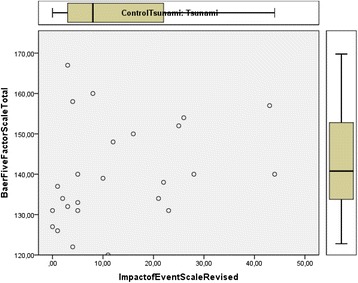
Scatterplot of association between mindfulness and posttraumatic stress in the disaster group (*n* = 25)

The statistical analysis was done with IBM SPSS Statistics 22. A scatterplot was drawn with total FFMQ and IES-R as coordinates (Fig. 1). The mean scores for the total FFMQ and the sub-facets of MF were calculated (Table 2). A linear regression with adjustments for gender, age, and years of education (Table 3) was applied to examine the associations between the total FFMQ scores and posttraumatic stress symptoms (total IES-R). We adjusted for the variables of gender, age and years of education as these variables are commonly adjusted for in studies. We used step-wise linear regression in SPSS in order to examine whether our results were related to these variables. Linear regression showed that none of these three variables were confounders. Possible linear correlations were examined between the total FFMQ including the sub-facets of observing, describing, acting with awareness, non-judging and non-reacting, and the IES-R including the sub-factors of intrusion, avoidance and hyper-arousal (Table 4).

**Table 2:** Mean scores for the Five-Factor Mindfulness Questionnaire sub-scales of observing, describing, acting with awareness, non-judging, and non-reacting (standard deviations in parentheses) and the total scores for the disaster-exposed group

	Disaster group (*n* = 25)
FFMQ Observing	27.76 (5.75)
FFMQ Describing	29.04 (4.50)
FFMQ Acting with Awareness	29.12 (5.07)
FFMQ Non-judging	32.72 (3.55)
FFMQ Non-reacting	21.40 (2.77)
FFMQ Total	140.04 (12.54)

**Table 3:** Association between mindfulness and posttraumatic stress in the disaster group (*n* = 25)

	Disaster group			
	B	R-square	95 % CI	*p*-value
FFMQ Unadjusted	.302	.086	-.122 – .726	.155
FFMQ Adjusted	.172	.046	-.382 – .726	.524

The unadjusted results and the results after adjustment for gender, age, and years of education are shown. B refers to the unstandardized regression coefficient

*FFMQ* five-factor mindfulness questionnaire

**Table 4:** Linear correlations (Pearson correlation coefficient) with *p*-values in parentheses, for mindfulness and posttraumatic stress including the five Mindfulness Questionnaire sub-scales of observing, describing, acting with awareness, non-judging, and non-reacting as well as the three Impact of Event sub-scales of intrusion, avoidance and hyperarousal

	IES Total	FFMQ Total	FFMQ Obs	FFMQ Descr	FFMQ Aware	FFMQ Non-judge	FFMQ Non-react	IES Intrusion	IES Avoid	IES Hyper arousal
IES Total	1	.293 (.155)	.313 (.127)	.439* (.028)	.033 (.877)	-.285 (.167)	.270 (.192)	.649** (.000)	.396* (.050)	.591** (.002)
FFMQ Total		1	.654** (.000)	.830** (.000)	.532** (.006)	.243 (.241)	.535** (.006)	.131 (.531)	.162 (.440)	.112 (.595)
FFMQ Obs			1	.615** (.000)	-.176 (.400)	-.175 (.404)	.433* (.031)	.110 (.600)	.185 (.377)	.079 (.707)
FFMQ Descr				1	.326 (.111)	-.132 (.529)	.430 (.032)	.178 (.396)	.082 (.698)	.177 (.397)
FFMQ Aware					1	.307 (.135)	.020 (.924)	.051 (.809)	.101 (.630)	.051 (.890)
FFMQ Non-judge						1	-.166 (.428)	-.232 (.265)	-.082 (.698)	-.206 (.322)
FFMQ Non-react							1	.282 (.172)	.135 (.518)	.225 (.281)
IES Intrusion								1	.715** (.000)	.900** (.000)
IES Avoid									1	.608** (.001)
IES Hyper arousal										1

*n* = 25

*correlation is significant at the 0.05 level

**correlation is significant at the 0.01 level

## Results

Mean scores for the total FFMQ and the sub-facets of MF were calculated (Table 2).

Linear regression analysis revealed no significant relationship between the FFMQ and IES-R scores (Table 3).

A scatterplot for the association of mindfulness and posttraumatic stress is shown in Fig. 1. This plot shows no association between these variables, not even of a non-linear kind. Moreover, there were no significant linear correlations between posttraumatic stress and the mindfulness sub-facets of observing, acting with awareness, non-judging, and non-reacting (Table 4). However, there was a significant positive correlation between the descriptive factor of mindfulness and the IES-R total (Table 4). There were no significant linear correlations between the five sub-facets of mindfulness and the three categories of posttraumatic symptoms, intrusion, avoidance and hyper-arousal (Table 4).

## Discussion

In this study, we found no significant linear correlations between MF measured by the total FFMQ score and posttraumatic stress measured by the IES-R. Neither did we find any significant linear correlations between posttraumatic stress and the MF sub-facets of observing, acting with awareness, non-judging, and non-reacting. A scatterplot of the data did not indicate any non-linear associations between any of these variables. There was a significant positive correlation between the descriptive factor of MF and IES-R total. There were no significant linear correlations between the five sub-facets of MF and the three categories of posttraumatic symptoms, intrusion, avoidance and hyper-arousal. The hypothesis that MF serves as a protective factor against the symptoms of posttraumatic stress disorder and that MF and posttraumatic stress are negatively related were not supported in this study.

The unexpected positive correlation between amount of posttraumatic stress symptoms and the describing aspect of MF might be related to an individual’s ability to describe his/her feelings as this is what the descriptive sub-facet of MF, measured by the FFMQ, stands for. Concerning this sub-facet of MF, our results mean that the bigger amount of posttraumatic stress symptoms an individual reports, the higher the ability to describe one’s feelings. Intervening variables, such as cognitive capacity, might affect this relation. Although this finding is not intuitively in line with the aspects of avoidance and hyper-arousal in posttraumatic stress, our findings might be related to the fact that our sample, on group level, was a standard deviation above average intelligence (Table 1) and had opted for participation in the study. Individuals with more severe posttraumatic stress symptoms of avoidance and hyper-arousal might have been more reluctant to participate in the study. Thus selection bias might be a factor affecting the result of positive correlation between the descriptive aspect of MF and the amount of posttraumatic stress.

The results of our study are in contrast to those of the study by Garland and Roberts-Lewis [12], which reported that dispositional MF appears to be a protective factor that buffers individuals against the experience of severe posttraumatic stress. Our study does not support the hypothesis that MF serves as a protective factor against the symptoms of posttraumatic stress disorder and that MF and posttraumatic stress are negatively related. The participants in the Garland and Roberts-Lewis study had extensive trauma histories and psychiatric symptoms. In our study, the participants were cognitively well-functioning, highly educated individuals with no or very few psychiatric symptoms. The individuals in our disaster sample did not have histories of complex or extensive trauma; rather, these individuals had suffered a single, albeit devastating, trauma during their first-hand exposure to the 2004 tsunami in Khao Lak, Thailand.

Our results can be compared with the Vujanovic et al. study [9], in which only small or moderate correlations between trait MF and posttraumatic stress were found in individuals without axis I psychopathologies. The results of our study, which revealed no significant negative correlations between trait MF and symptoms of posttraumatic stress, were based on a non-clinical disaster-exposed sample as well, and may or may not have been different if we had examined a clinical sample. Thus, we stress the importance of replication studies that investigate the relationship between MF and posttraumatic stress in clinical and non-clinical samples.

In their review article, Thompson et al. [11] suggested that trait MF is associated with greater psychological adjustment following exposure to trauma; furthermore, experiential avoidance, persistent dissociation, and coping strategies involving emotional disengagement are associated with greater posttraumatic stress severity and related psychopathologies. It should be noted that in addition to MF, many factors may be associated with greater psychological adjustment following trauma exposure, such as the type of trauma, trauma history, presence of psychiatric symptoms, and level of cognitive functioning. These factors may, in turn, affect coping strategies, self-regulation, and emotional regulation in different ways. Therefore, we wish to underline that in models examining the relationship between MF and posttraumatic stress, factors such as the type of trauma, trauma history, psychiatric symptomatology, and the cognitive and self-regulatory strategies of the individual should be taken into account. It is possible that MF may not be a protective factor against posttraumatic stress in situations in which the self-regulatory strategies of the trauma-exposed individual are low because MF implies an exposure component of memories, affects, and thoughts. It is also possible that exposure to trauma may affect attentional functioning such that the potential protective quality of MF against posttraumatic stress becomes compromised. Posttraumatic hyper-vigilance may counteract the non-judging and non-reacting aspects of MF and thus interfere with the ability of MF to act as a protective factor against the symptoms of posttraumatic stress. We wish to underline that although we found no significant associations between trait MF and posttraumatic stress in this study, MF-based treatments in trauma-adapted formats may still be beneficial for trauma-exposed individuals with symptoms of posttraumatic stress.

Our study has some limitations that should be noted. The sample size was small, and the design was cross-sectional, which precludes causal interpretations. Our results may also be affected by the fact that only less than half of the tsunami-exposed individuals in Norway agreed to participate in this study. The fact that a few years had passed since the disaster exposure might affect the study results. The results of this study imply that the possible associations between different aspects of MF and posttraumatic stress symptoms need to be examined. This study should be replicated in larger samples of different trauma-exposed clinical and non-clinical populations. We consider the factor of trauma history to be important. Our study was based on a single trauma; the results may vary between groups that have been exposed to single versus complex and repeated traumas. The types of trauma (e.g., single, complex, extensive, or ongoing trauma) and the resources of the individuals (e.g., coping abilities, cognitive functioning, psychiatric symptoms, and self-regulatory strategies) need to be accounted for in future studies.

## Conclusions

Our findings do not indicate a relationship between dispositional mindfulness and posttraumatic stress levels after exposure to a trauma, except for the descriptive sub-facet of mindfulness and here the correlation is positive and not negative as would be expected if mindfulness is a protective factor for posttraumatic stress. Future studies should investigate the relationship between mindfulness and posttraumatic stress while accounting for factors such as trauma history, type of trauma, and individual differences in traumatic stress reactions.

## Abbreviations

FFMQ, the five-facet mindfulness questionnaire; IES-R, the impact of event scale – revised version; MF, mindfulness; PCL, the posttraumatic stress disorder checklist; WASI, Wechsler Adult Short Intelligence Test
